# The expression and association of MATK in chronic hypoperfusion patients with white matter hyperintensity

**DOI:** 10.3389/fnagi.2025.1570482

**Published:** 2025-08-26

**Authors:** Ming Zhao, Jiayue Zhang, Qianqian Yang, Lei Chen, Hongjian Shen, Yingyan Pu, Wei Wei, Yi Han, Ping Zhang

**Affiliations:** ^1^Department of Neurology, Naval Medical Center of PLA, Naval Medical University, Shanghai, China; ^2^Department of Neurology, Shenzhen Samii International Medical Center (The Fourth People’s Hospital of Shenzhen), Shenzhen, China; ^3^Department of Obstetrics and Gynecology, Changhai Hospital, Naval Medical University, Shanghai, China; ^4^Department of Cerebrovascular Disease Center, Changhai Hospital, Naval Medical University, Shanghai, China; ^5^Institute of Neuroscience, Key Laboratory of Molecular Neurobiology of the Ministry of Education and the Collaborative Innovation Center for Brain Science, Naval Medical University, Shanghai, China

**Keywords:** chronic cerebral hypoperfusion, white matter hyperintensity, magnetic resonance imaging, MATK, vascular cogitive impairment

## Abstract

**Background:**

Chronic cerebral hypoperfusion (CCH) is a key contributor to vascular cognitive impairment (VCI). Patients with CCH frequently exhibit white matter hyperintensities (WMH) on MRI. This study investigated the expression and clinical relevance of megakaryocyte-associated tyrosine kinase (MATK) in CCH patients with WMH.

**Methods:**

We recruited 42 anterior circulation stenosis patients with CCH and WMH (CCH-WMH group) and 45 age-matched healthy controls. MATK mRNA expression in peripheral blood mononuclear cells (PBMCs) was quantified using qPCR. WMH severity was graded using the Fazekas scale. Correlations between MATK expression and imaging/clinical parameters were analyzed using Pearson’s correlation and logistic regression. Diagnostic performance was assessed via ROC analysis.

**Results:**

Megakaryocyte-associated tyrosine kinase expression was significantly downregulated in the CCH-WMH group versus controls (1.20 ± 0.99 vs. 1.84 ± 0.87; *P* < 0.01). MATK levels showed a strong negative correlation with Fazekas scores (R^2^ = 0.3405, *P* < 0.001). Multivariate regression identified MATK as independently associated with WMH (aOR = 0.492; 95% CI: 0.262–0.923; *P* = 0.027). ROC analysis demonstrated moderate diagnostic accuracy for WMH (AUC = 0.743; 95% CI: 0.637–0.849).

**Conclusion:**

Reduced peripheral MATK expression correlates with WMH severity in CCH patients and may serve as a potential diagnostic biomarker.

## Highlights

- Patients with WMH had worse MoCA scores;
- MATK was downregulated in the WMH group;
- MATK exhibited a significant negative correlation with the degree of WMH;
- MATK was independently associated with WMH in patients with CCH.

## Introduction

Vascular cognitive impairment (VCI) is one of the most important causes of cognitive disorders in the elderly, accounting for 20%–40% of dementia diagnoses, second only to Alzheimer’s disease (AD), and seriously affecting the quality of life of patients (Rundek et al., 2022; Wallin et al., 2013). Cerebral hypoperfusion can contribute to VCI, especially chronic cerebral hypoperfusion (CCH), which has a significant correlation with the severity of dementia and predicts which individuals with mild cognitive impairment (MCI) will progress to dementia (Alsop et al., 2010; Chao et al., 2010).

This pathological change is due to the cerebral deep vessels of white matter being more vulnerable to ischemia and hypoxia. Therefore, CCH can cause ischemic changes in the white matter, leading to the destruction of the blood-brain barrier and inflammatory cell infiltration, resulting in demyelination in the white matter of the brain. These lesions can be detected by magnetic resonance imaging (MRI) with white matter hyperintensities (WMH) on the T2-weighted fluid-attenuated inversion recovery (T2-FLAIR) sequence (Barker et al., 2014; Bastin et al., 2009; Fernando et al., 2006; ten Dam et al., 2007). Common causes of cerebral hypoperfusion include carotid stenosis, intracranial atherosclerosis, heart failure, and other diseases that cause abnormal cerebral blood flow dynamics (Hamaguchi et al., 1996; Kim et al., 2004). However, there is still no clear evidence on whether patients with CCH will develop WMH and cognitive dysfunction, and there is a lack of specific biomarkers to predict the prognosis of the disease.

Megakaryocyte-associated tyrosine kinase (MATK), also known as Csk homologous kinase (CHK), is a non-receptor tyrosine kinase of the Csk family, primarily expressed in blood cells and the brain. MATK can be found in both astrocytes and oligodendrocytes and is related to the differentiation of oligodendrocytes (Hamaguchi et al., 1996). Overexpression of MATK in neuroblastoma and astrocytoma cells can inhibit the growth and differentiation of tumor cells (Kim et al., 2004). Studies have shown that MATK can promote the differentiation of anti-inflammatory monocytes. Cytokines IL-4 and its family can increase the expression of MATK in peripheral blood monocytes, while IFN-γ reduces its expression (Hiremath et al., 2004). Inflammatory monocytes are significantly correlated with the progression of cerebrovascular disease, leading to an increase in WMH volume (Noz et al., 2018). However, the role of MATK in CCH patients with WMH has not been reported. The purpose of this study was to analyze the correlation between WMH severity and serum MATK expression in patients with CCH.

## Materials and methods

### Study population

The WMH group consisted of 42 CCH patients with WMH from the Changhai Hospital from February 2022 to February 2023, including 24 males and 18 females, with an average age of 67.74 ± 4.85 years. CCH was defined as the reduced perfusion in one cerebral hemisphere detected by computed tomography perfusion (CTP) with severe middle cerebral artery (MCA)/internal carotid artery (ICA) stenosis; the contralateral cerebral hemisphere of the same patient was set as the control. WMH was defined as hyperintense lesions on T2-FLAIR according to the Standards for Reporting Vascular Changes on Neuroimaging (STRIVE) criteria. Inclusion criteria were: (1) reduced perfusion in one or both cerebral hemispheres detected by CTP for > 6 months; (2) with severe MCA or ICA stenosis; (3) white matter hyperintensities on the T2-FLAIR sequence of MRI. Exclusion criteria were: (1) cognitive decline due to neurological diseases (e.g., Alzheimer’s disease, vascular dementia, and Parkinson’s disease dementia); (2) hereditary dementia; (3) depression or mental illness; (4) history of acute stroke, intracranial infection, or traumatic brain injury; (5) blood diseases, tumors, and other systemic diseases. The control group included 45 age-matched healthy volunteers with normal MRI findings, including 23 males and 22 females, with an average age of 63.84 ± 5.78 years. Demographic characteristics and Montreal Cognitive Assessment (MoCA) scores of patients were collected. The study protocol was approved by the Ethics Committee of the Changhai Hospital and performed in compliance with the Code of Ethics of the World Medical Association (Declaration of Helsinki). All participants provided written informed consent before participation.

### Cognitive assessment

Cognition was evaluated using the MoCA test, which includes naming, attention, language, abstraction, memory, and orientation. Data were normalized with age and education-matched norms, and cognitive deficit was determined by a score of <26. This test is widely accepted for screening mild cognitive impairment based on Chinese population (Chen et al., 2016; Liu et al., 2022). To comprehensively evaluate the cognitive domain, we employed established instruments validated for VCI diagnosis: the Auditory Verbal Learning Test Delayed Recall (AVLT-DR), Trail Making Test Parts B (TMT-B), Symbol Digit Modalities Test (SDMT) to assess working memory capacity (Li et al., 2022; Skrobot et al., 2018).

### Isolation of mononuclear cells and MATK expression by qPCR

Peripheral blood mononuclear cells (PBMCs) were isolated from human peripheral blood using a lymphocyte separation medium (GE Healthcare) as described previously (Skrobot et al., 2018). Total RNA was extracted from PBMCs using Trizol RNA isolation reagent (Invitrogen) according to the manufacturer’s protocol. cDNA from each sample was synthesized using a RevertAid First Strand cDNA Synthesis Kit (Thermo Scientific). qPCR was performed on a LightCycler 96 apparatus (Roche) using the SYBR Green Real-time PCR Master Mix (Toyobo). Expression of GAPDH in cDNA samples was used to control for differences in the extraction and reverse transcription of total RNA. Gene expression was normalized to a standard housekeeping gene using the ΔΔCT method.

### The following primers were used for real-time RT-PCR

MATK 5′-GGTGAGACCAAAGCGGAAACAC-3′ (sense); 5′- TCCGATCTGTGCTCCCAATGTC-3′ (antisense); GAPDH 5′-AA ATGGTGAAGGTCGGTGTG-3′ (sense); and 5′-AGGTCAATGA AGGGGTCGTT-3′ (antisense).

### Imaging acquisition

All patients underwent MRI on a 3.0-T MRI system (GE Signa 3.0 T HDxt, GE Healthcare, WI, USA) using an eight-channel head coil. The protocol included T1-weighted (T1WI), T2-weighted (T2WI), and T2-FLAIR sequences. The parameters were as follows: (1) T2 FLAIR: TR/TE = 8000/97.0 ms, FOV = 220 × 220 mm^2^, slice thickness = 5 mm, slice gaps = 1.5, flip angle = 150.0, ETL = 19, matrix = 320 × 420; (2) T2-weighted FSE: TR/TE = 2883/50 ms, FOV = 10 × 10 cm^2^, NEX = 3, matrix = 320 × 256, echo-train length (ETL) = 20, slice thickness = 2 mm, and sequence duration = 111 s, no-phase-wrap option (acquiring a larger FOV and cut the edges) was used to avoid wrapping artifacts. (3) T1WI: TR/TE = 567/16 ms, FOV = 10 × 10 cm^2^, NEX = 2, matrix = 320 × 256, ETL = 6, slice thickness = 2 mm, and sequence duration = 48.4 s. WMHs were grouped according to the distribution of hyperintensities in the paraventricular and deep white matter on T2WI using the Fazekas scale (0, normal; 1, mild; 2, moderate; 3, severe). All MRI scans were independently assessed by two board-certified neuroradiologists (8 and 11 years of experience) blinded to clinical grouping, biomarker data, and patient histories. Inter-rater reliability was quantified using intraclass correlation coefficients (ICC) for continuous variables and Cohen’s κ for categorical ratings, with discrepancies resolved through consensus review involving a third senior neuroradiologist.

### Statistical analysis

All statistical analyses were performed using SPSS24.0 and GraphPad Prism 8.0. First, the test of normal distribution was conducted on all metrics. Then, mean and standard deviation (SD) were recorded for continuous variables, and frequency and percentage were recorded for categorical variables. Student’s *t*-test was used to compare continuous variables between the two groups. Chi-square test was used for categorical variables. Pearson correlation analysis was used to assess the relationship between MATK levels and Fazekas scales. Binary logistic regression was employed to examine cross-sectional associations between MATK levels and WMH. Multivariable models adjusted for age, sex, hypertension, diabetes, smoking, drinking and MoCA status. Receiver operating characteristic (ROC) analysis evaluated the diagnostic accuracy of MATK in discriminating between CCH patients with or without WMH at the time of assessment. Statistical significance was set at *P* < 0.05 (two-tailed).

## Results

### Cerebral MRI examination

**FIGURE 1:**
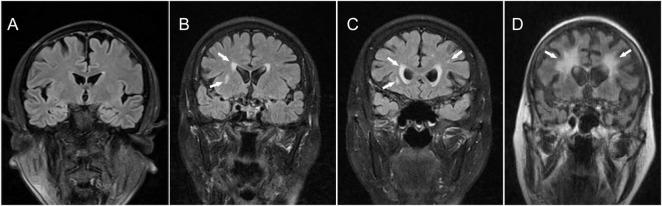
Magnetic resonance imaging examination of the brain. **(A)** Grade-0 represents normal or non-dots WMHs; **(B)** Grade-1 presents multiple dots WMHs; **(C)** Grade-2 is a fusion lesion with punctured WMHs junctions, which can also be found in deep white matter; **(D)** Grade-3 showed extensive fusion of WMHs with brain atrophy. White arrows represent WMHs.

As shown in Figure 1, MRI monitoring revealed that patients in the WMH group showed various cerebral WMH, especially near the lateral ventricles. We graded patients on a scale of 0–3 according to the extent of fusion of WMH by the Fazekas scale. The white matter of patients with CCH appears hypointense on T1 weighted, but punctate, patchy, or fusion areas of hyperintensities on T2 weighted, in addition to hyperintensity changes in the lateral ventricles, the deep white matter was also found WMHs in patients with severe CCH (Figures 1B, C). As white matter lesions worsen, CCH patients with WMH also developed varying degrees of brain atrophy (Figure 1D). This result also verified that cerebral white matter damage caused by cerebral chronic hypoperfusion is a characteristic manifestation of developing cerebral degenerative pathology, which may cause cognitive impairment.

### WMH patients and healthy controls demographics, and cognitive function characteristics

Magnetic resonance imaging data from 42 WMH patients (63.84 ± 5.78 years old; 18 females) and 45 healthy controls (67.74 ± 4.85 years old; 22 females) were eventually included in the study and analyzed. The control group without WMH was randomly selected, the baseline data and Fazekas scales of the two groups were recorded, and the MoCA score was used for rapid cognitive function assessment. The WMH patient demographics are listed in Table 1. It can be seen that the WMH differed significantly from the control group among the elderly (67.74 ± 4.85 years versus 63.84 ± 5.78 years; *P* = 0.010), there was no difference in the gender ratio (40% versus 52.38% females; *P* = 0.668). Regarding comorbidities, patients with WMH more frequently had hypertension (60% versus 38.1%; *P* = 0.010), although the proportion of diabetes mellitus (37.78% versus 35.31%; *P* = 0.513) and hyperlipidemia (42.22% versus 33.33%; *P* = 0.192) were higher, there was no statistical difference.

**TABLE 1:** Demographics characteristics between WMH patient and healthy control.

Clinical characteristics	WMH patients (*N* = 42)	Control (*N* = 45)	*P*-value
Age	67.74 ± 4.85	63.84 ± 5.78	**0.001**
Gender			
Female	18 (40%)	22 (52.38%)	0.668
Hypertension	27 (60%)	16 (38.10%)	**0.010**
Diabetes mellitus	17 (37.78%)	15 (35.71%)	0.513
Hyperlipidemia	19 (42.22%)	14 (33.33%)	0.192
Smoking	13 (28.89)	10 (23.81%)	0.467
Drinking	9 (20%)	8 (19.05%)	0.789
Cerebral artery stenosis (ICA/MCA)			**<0.001**
<70%	11 (26.19%)	0	
70%–90%	22 (52.38%)	0	
>90%	9 (21.43%)	0	
Fezakas	1.833 ± 0.82	0	**<0.001**
Education	9.36 ± 3.18	10.02 ± 3.26	0.466
MOCA	24.36 ± 2.31	26.11 ± 1.87	**<0.001**
TMT-B	135.45 ± 31.11	89.8 ± 23.31	**<0.001**
SDMT	25.8 ± 6.35	33.28 ± 8.09	**<0.001**
AVLT	10.8 ± 4.32	18.36 ± 2.97	**<0.001**

WMH, White Matter Hyperintensity; ICA, Internal carotid artery; MCA, Middle cerebral artery; MOCA, Montreal Cognitive Assessment; TMT, Trail Making Test Parts B; SDMT, Symbol Digit Modalities Test; AVLT, Auditory Verbal Learning Test. The bold value means *p* < 0.05.

The average Fazekas scale of WMH patients was1.833 ± 0.82, which had significant differences compared to healthy controls (*P* < 0.010). After adjustment of education, patients with WMH had worse MoCA scores (24.36 ± 2.31 versus 26.11 ± 1.87; *P* < 0.010), suggesting a more severe cognitive impairment. To address the multidimensional nature of VCI, we supplemented MoCA with domain-specific neuropsychological tests. The WMH group demonstrated significantly poorer performance compared to the Control group on the SDMT and AVLT (all *P* < 0.001). Conversely, the WMH group exhibited significantly higher (worse) scores on TMT-B (*P* < 0.001). These findings indicate significant impairments in executive function, processing speed, and episodic memory within the WMH group.

### The expression of MATK mRNAs in mononuclear cells of peripheral venous blood

**FIGURE 2:**
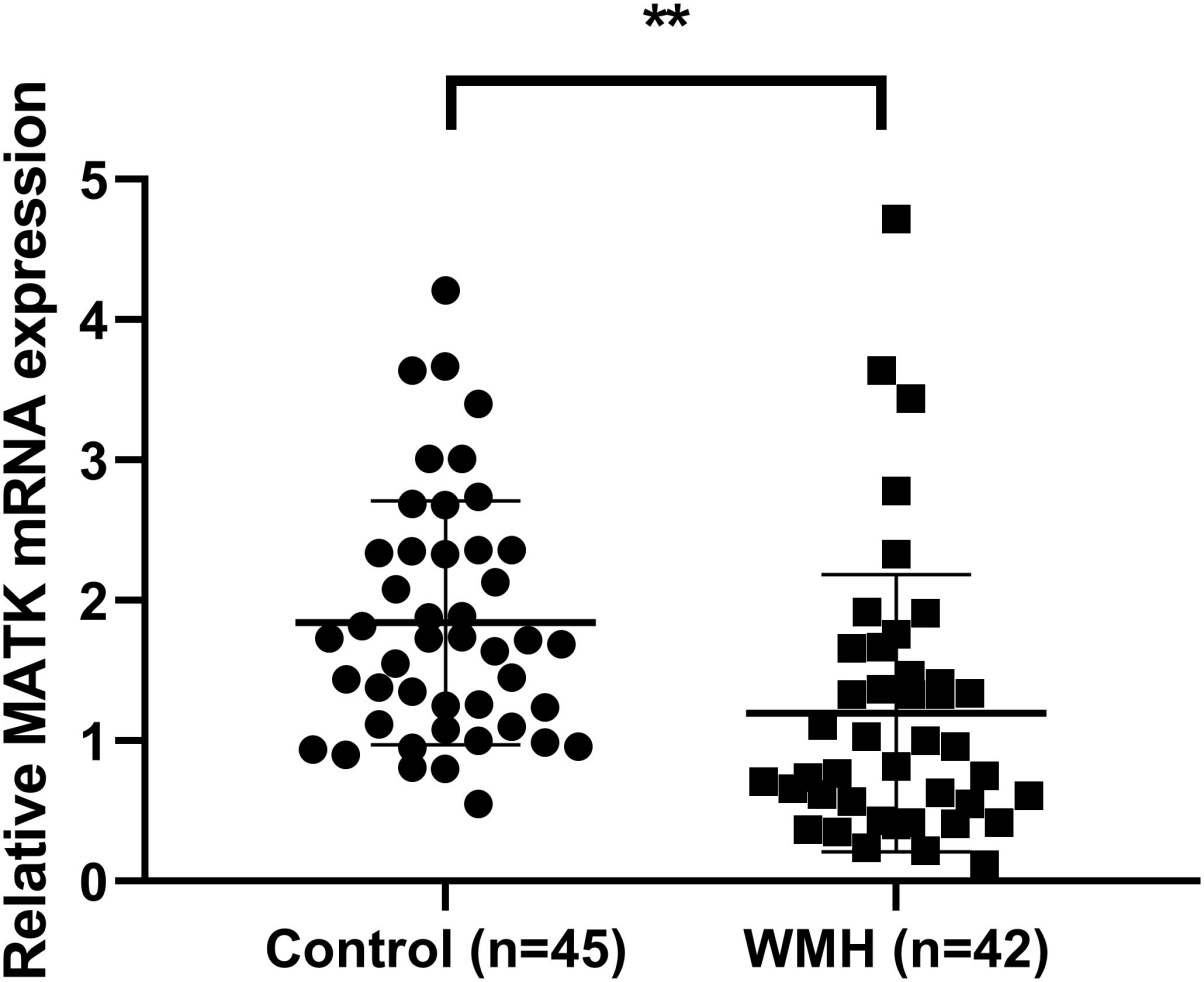
The expression of MATK in the mononuclear cells from peripheral blood of patients with WMH. PBMCs were isolated from human peripheral blood by gradient separation, and qPCR was used to detect the relative expression of MATK in mononuclear cells. **P < 0.01 compared with the control group.

Because mononuclear cells are critical in the pathogenesis of WMH, we next explored the correlation between MATK in mononuclear cells and WMH. We first examined the MATK mRNAs expression in mononuclear cells sorted by lymphocyte separation medium of peripheral venous blood from the WMH and control group. The qPCR results showed that the relative MATK expression was downregulated in the WMH group compared to the control group (Figure 2). The expression of MATK in the control group was 1.84 ± 0.87, while the expression of MATK in the WMH group was 1.20 ± 0.99, which was a statistically significant difference (*t* = 3.24, *P* < 0.01). The data suggest that MATK in the PBMCs may be a protective factor for WMH patients.

### Correlation analysis between WMH and MoCA with MATK

**FIGURE 3:**
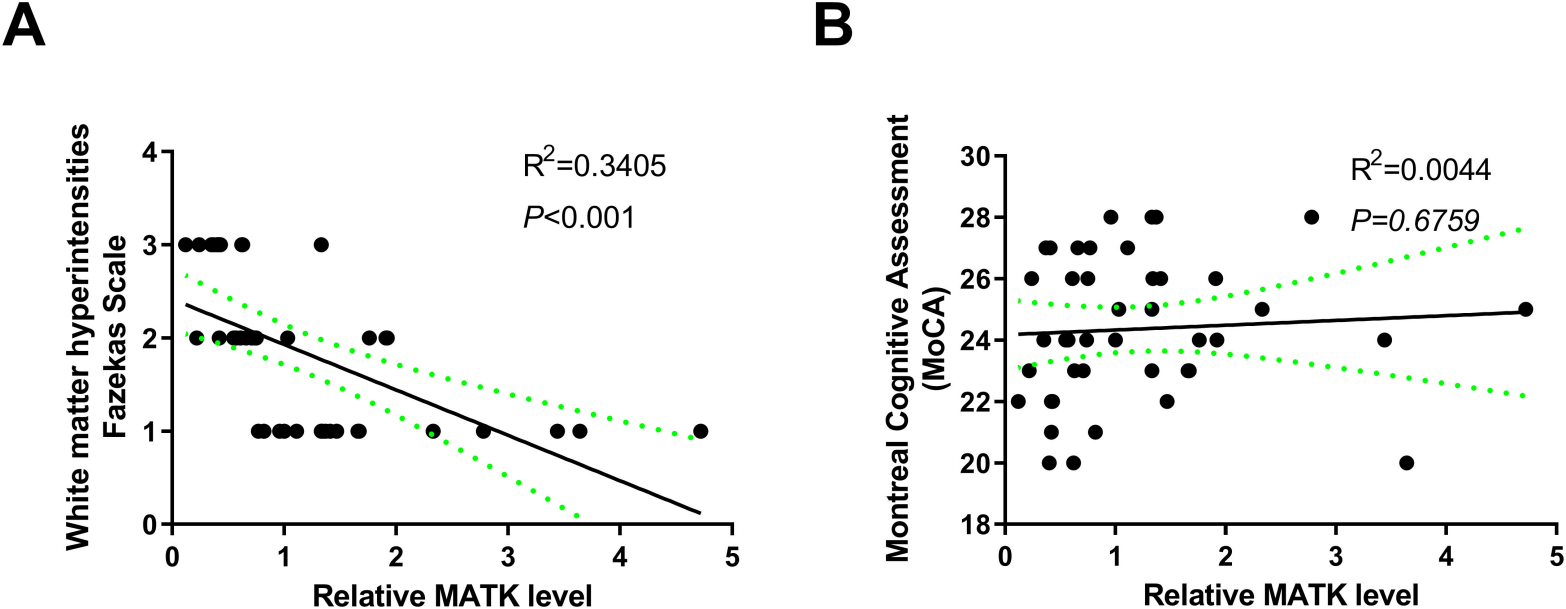
Correlation of MATK with MoCA and WMH. **(A)** Pearson’s analysis of the correlation between MATK and the Fazekas Scale. **(B)** Pearson’s analysis of the correlation between MATK and MoCA.

To further verify whether MATK expression in peripheral blood could reflect WMH severity *in vivo*, we then assessed the correlation between the relative expression of MATK in peripheral blood and Fazekas scales using Pearson’s correlation analysis and found that as the MATK decreased, the Fazekas scales gradually raised (R^2^ = 0.3405, *P* < 0.001) (Figure 3A), indicating that changes in MATK expression exhibited a significant negative correlation with the degree of WMH. However, there was no significant correlation between MATK and MoCA score (R^2^ = 0.0044, *P* = 0.6759) (Figure 3B). Similarly, MATK showed no significant correlation with performance on domain-specific neuropsychological tests, including TMT, SDMT, and AVLT (Supplementary Figure 1).

### Relationship between MATK and the stenosis of cerebral artery in patients with WMH

Since the WMH patients we selected were all complicated with carotid artery stenosis or of middle cerebral artery stenosis along with cerebral hypoperfusion, we wanted to know the relationship between different arterial stenosis and MATK expression in CCH-WMH patients. So, we divided the WMH patients into the mild stenosis (<50%) group, the moderate stenosis (50%–70%) group and the severe stenosis (>70%) group according to different degrees of stenosis. It was found that in the WMH group, the expression of MATK in the group with cerebral artery stenosis <50% (26.2%) was significantly higher than that in the 50%–70% (52.4%) and >70% (21.4%) groups (*P* < 0.001). There was no significant difference between 50%–70% and >70% (*P* = 0.4770), indicating that the expression of MATK decreased in patients with CCH-WMH complicated with moderate to severe cerebral artery stenosis (>50%) (Supplementary Figure 2).

### The association between MATK and WMH with CCH in anterior circulation cerebral artery stenosis patients

Univariate logistic regression analysis showed that age, hypertension, MoCA, and MATK were also associated with WMH (Table 2). Multivariate analysis showed that MATK levels (adjusted OR 0.492 [95% CI, 0.262–0.923], *P* = 0.027) were independently associated with WMH in patients with CCH adjusted for age, female, hypertension, diabetes mellitus, hyperlipidemia, smoking and drinking.

**TABLE 2:** The association between MATK and WMH with CCH in anterior circulation cerebral artery stenosis patients.

Clinical characteristics	cOR (95% CI)	*P*-value	aOR (95% CI)	*P*-value
Age	1.154 (1.051–1.266)	**0.003**	1.114 (0.994–1.250)	0.064
Female	1.275 (0.547–2.971)	0.573		
Hypertension	3.262 (1.356–7.850)	**0.008**	1.690 (0.589–4.851)	0.329
Diabetes mellitus	1.360 (0.568–3.259)	0.490		
Hyperlipidemia	1.829 (0.762–4.393)	0.177		
Smoking	1.103 (0.440–2.765)	0.834		
Drinking	1.091 (0.386–3.079)	0.869		
MOCA	0.675 (0.539–0.846)	**0.001**	0.777 (0.602–1.005)	0.054
MATK	0.447 (0.259–0.771)	**0.004**	0.492 (0.262–0.923)	**0.027**

cOR, crude odds ratio; CI, confidence interval; aOR, adjusted odds ratio; WMH, White Matter Hyperintensity; MOCA, Montreal Cognitive Assessment. The bold value means *p* < 0.05.

**FIGURE 4:**
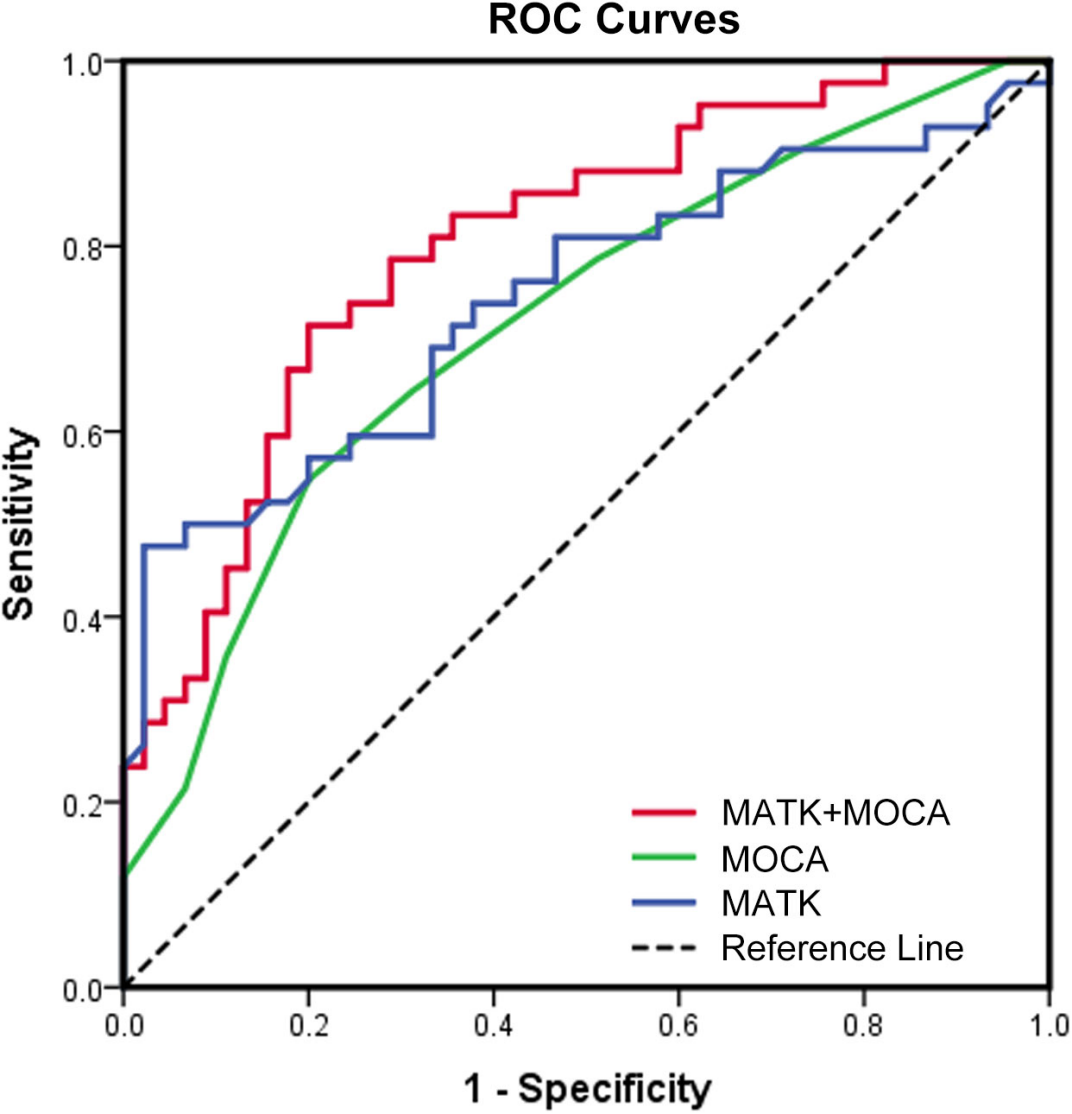
Receiver operating characteristic (ROC) curves on the basis of binary logistic regression. ROC, receiver operating characteristic.

We also generated ROC curves to explore the diagnostic values for WMH. Among them, the area under curve (AUC) value of the final regression model (including both of the independent risk factors) was 0.803 (*P* < 0.001) and had the highest diagnostic accuracy. The AUCs for MATK was 0.743 (95% CI 0.6369–0.8493, *P* < 0.001). ROC analysis demonstrated MATK’s accuracy in discriminating concurrent WMH from no WMH alone were demonstrated with a cutoff value of 0.454, a sensitivity: 0.4762, and a specificity: 0.9778 (Figure 4).

## Discussion

Cerebral hypoperfusion is one of the causes that contribute to the occurrence and development of VCI and dementia. With the decrease in cerebral blood flow (CBF), the cognitive function of patients also deteriorates (Ferro et al., 2020; Román, 2004). This reduction in CBF is related to the severity of WMHs under T2 sequences of MRI (Bivard et al., 2016; Wong et al., 2019). However, the pathological mechanisms of hypoperfusion-induced white matter damage remain incompletely elucidated, and there are still no effective diagnostic methods to predict the occurrence of WMH. In the present study, we investigated the expression of MATK in peripheral blood and verified whether MATK could be a biomarker for predicting and diagnosing WMH and VCI.

Studies have shown that CCH, like cerebral small-vessel disease (CSVD), was often ignored by patients due to less severe clinical symptoms or lower mortality than common stroke. With the development of neuroimaging equipment, MRI can allow us to detect the presence of CSVD at an early stage. Similarly, we can prevent the progression of CCH through effective treatment measures. MRI examination in elderly patients with cognitive dysfunction shows that WMH is one of the biomarkers for the diagnosis of CSVD (Debette and Markus, 2010; Gouw et al., 2011; Prins and Scheltens, 2015). MRI scans of 560 older individuals, the subjects were followed up with annual neuropsychological examinations for 3 years and evaluation of instrumental activities of daily living for 7 years, found that WMH was an independent predictor of cognitive impairment in a multivariate linear mixed model (Jokinen et al., 2020). However, just relying on WMH is not specific for the diagnosis of CSVD, because it is difficult to distinguish CSVD or CCH from other demyelinating diseases such as multiple sclerosis and optic neuromyelitis by WMHs (Pantoni, 2010). There was also some mismatch between WMH and cognitive function scores in patients with CSVD. Among 156 patients with CSVD, 37 (23.7%) showed mismatching. Conventional imaging features and penetrating artery injury may explain this heterogeneity (Wang et al., 2021). Therefore, the use of other biomarkers to further clarify the prognosis of CCH is particularly important. In patients with a clinical diagnosis of CSVD, changes in several biomarkers such as low molecular weight neurofilament (NF-L), matrix metalloproteinase-9 (MMP-9), tissue inhibitor of metalloproteinase-1 (TIMP-1), matrix metalloproteinase-2 (MMP-2) and albumin CSF/plasma ratio were found to be increased (Corriveau et al., 2017; Swardfager et al., 2017; Wallin et al., 2017). In our study, we found that the relative mRNA expression of MATK in the peripheral blood of patients with CCH was significantly decreased, and it was significantly negatively correlated with Fazekas scales. Logistic regression analysis indicated that MATK was an independent factor strongly associated with WMH. The area under the ROC curve of MATK was 0.743, which was not significantly different from the final regression model 0.803, suggesting that MATK can be used as a biomarker for diagnosis of WMH, indicating that MATK can provide help in the diagnosis accuracy of CCH. While the Fazekas visual scale provided a clinically efficient WMH severity metric in this study, yet lacks volumetric granularity for diffuse non-confluent pathology. Crucially, our observed MATK-Fazekas association (R^2^ = 0.3405, *P* < 0.001) specifically manifested in confluent WMH (Grade 2–3), affirming its sensitivity to biologically significant injury. Future investigations should supplement visual grading with semi-automated volumetry to validate Fazekas-defined thresholds against quantitative standards. Such integration will bridge clinical relevance with analytical precision.

Beyond impaired cerebral blood flow, emerging evidence implicates blood-brain barrier (BBB) disruption, neuroinflammation, and systemic factors as parallel contributors to white matter vulnerability (Liddelow et al., 2017; Wardlaw et al., 2013; Zhang et al., 2020). In hypoperfused states, endothelial activation triggers BBB leakage, allowing extravasation of plasma-derived neurotoxins (e.g., fibrinogen) that directly demyelinate axons and activate microglia (Wong et al., 2019). Concomitantly, ischemic stress induces reactive astrogliosis and microglial proliferation, with pro-inflammatory cytokines (e.g., IL-1β, TNF-α) amplifying oligodendrocyte apoptosis (Liddelow et al., 2017). Notably, systemic inflammation often comorbid with vascular risk factors, which may synergistically exacerbate this cascade through circulating leukocyte infiltration and glial priming (Low et al., 2019).

Megakaryocyte-associated tyrosine kinase is mainly expressed in blood cells and the brain (Noz et al., 2018), suggesting its potential role in regulating neuroinflammatory responses to hypoperfusion It has been reported that IL-4 can induce MATK/CHK expression in human peripheral blood mononuclear cells, while IFN-γ can inhibit it (Hiremath et al., 2004). Another study showed that mouse primary microglia cells were stimulated with IL-4 and IFN-γ respectively to obtain differential gene expression profiles of microglia cells *in vitro*. The expression of the MATK molecule was significantly up-regulated under IL-4 (M2 polarization) stimulation, but significantly down-regulated under IFN-γ (M1 polarization) stimulation (Yu et al., 2015). These studies suggest that MATK modulates anti-inflammatory responses in myeloid cells both in the peripheral circulation and the central nervous system, with its expression significantly increased in the anti-inflammatory environment.

Microglia cells are also one of the mechanisms that cause WMH in the brain of patients with CSVD. As resident macrophages of the central nervous system (CNS), microglia cells play the role of endogenous innate immunity. Although microglia cells are generally favorable initially in performing immune functions, prolonged or excessive activation can lead to cytotoxic effects (Luo et al., 2010; McGeer and McGeer, 2001). When chronic hypoperfusion and blood-brain barrier destruction occur in the brain, the infiltration of peripheral mononuclear macrophages and overactivation of microglia will aggravate the injury of white matter and cause the demyelination of axons. This is one of the important mechanisms of CCH brain injury.

While MATK has known roles in monocyte and glial cell function, this study only measures it at peripheral level. Its relevance to microglial phenotype remains speculative without brain tissue, CSF, or imaging correlates. In our study, we found that MATK expression was significantly decreased in the peripheral blood of patients with severe degrees of cerebral WMHs, so we hypothesis that reduced MATK can drive macrophages to the IFN-γ phenotype in the peripheral blood, which has an inflammatory damaging function. While within the brain, it may also promote microglia toward a pro-inflammatory phenotype to damage the white matter. But the role of MATK on the phenotypic conversion of microglia in CNS still needs further studies to be clarified.

White matter hyperintensities is very common in older adults and develops with age (Jorgensen et al., 2018). Black race, female sex, and the presence of the Apolipoprotein E (ApoE)-4 allele were all associated with greater WMH burden or progression (Brickman et al., 2014; Jorgensen et al., 2018). These factors are all non-modifiable factors as in hereditary CSVD, while the risk factors for WMH severity and progression are also associated with cerebrovascular, cardiometabolic, and nutritional (Jorgensen et al., 2018). Of these, hypertension had the strongest association. In a cross-sectional study, increased BP was clearly associated with the presence or severity of WMH. Hypertension is commonly found to be associated with the subsequent appearance of WMH in studies of early hypertension, with elevated blood pressure being able to increase the risk of developing WMH after 5 and 20 years. Similarly, midlife and older age BP were both associated with an increased risk of WMH (Vuorinen et al., 2015).In another study, increasing midlife BP was significantly associated with WMH volume in old age (Swan et al., 1998). A meta-analysis of the effects of antihypertensive drugs on CSVD showed that patients in the intensive BP-lowering group had significantly less WMH progression (van Middelaar et al., 2018).

The limitations of this study are as follows. First of all, our study design lacks a “CCH without WMH” group and a “WMH without CCH” cohort, which limits our ability to attribute MATK changes solely to CCH or WMH. Future studies should include these groups to clarify MATK’s specificity. And due to the limited number of cases collected, there may be some selective bias. It is necessary to expand the sample size in later studies to observe the overall change of MATK expression in CCH patients. Secondly, the CCH-WMH patients were accompanied by MCA/ICA artery stenosis, and intracranial artery stenosis itself is also a risk factor for WMH. Interestingly, our results also found a significant negative correlation between decreased MATK expression and cerebral artery stenosis, suggesting that the correlation between MATK and WMH may be related to cerebral artery stenosis. The role of MATK in the development of WMH can be determined in subsequent studies by observing whether intracranial artery stenosis alone causes changes in MATK expression. Third, MATK in peripheral blood is derived from monocytes and cannot fully represent the expression of MATK in intracranial microglia. Further animal experiments are needed to clarify the changes of MATK in the peripheral and brain pathology of WMH. As there are currently limited drugs that can treat WMH caused by CCH, the decreased expression of MATK in peripheral blood found in our study is a biomarker in CCH patients with WMH, so MATK may become a potential target for treating WMH in patients with CCH. Finding drugs that act on the MATK signaling pathway may be an effective way to treat the progression of WMH. As this is a cross-sectional study, causal relationships cannot be inferred. Longitudinal studies are needed to validate MATK’s prognostic value.

## Conclusion

Our cross-sectional data demonstrate that elevated MATK levels are independently associated with moderate-severe WMH burden in CCH patients. The discriminative accuracy (AUC = 0.78) suggests MATK may serve as a diagnostic biomarker for existing WMH severity.

## Data Availability

The raw data supporting the conclusions of this article will be made available by the authors, without undue reservation.

## Supplementary material

Supplementary Figure 1 Correlation of MATK with domain-specific neuropsychological tests. **(A)** Correlation of MATK with TMT-B. **(B)** Correlation of MATK with SDMT. **(C)** Correlation of MATK with SDMT. TMT, Trail Making Test Parts B; SDMT, Symbol Digit Modalities Test; AVLT, Auditory Verbal Learning Test.

Supplementary Figure 2 Correlation of MATK with WMH patients’ cerebral artery stenosis.
