# Supplementary figures and images for: The expression and association of MATK in chronic hypoperfusion patients with white matter hyperintensity
Source: Front Aging Neurosci. 2025 Aug 26;17:1570482. doi: 10.3389/fnagi.2025.1570482 (PMC12417534; doi:10.3389/fnagi.2025.1570482)

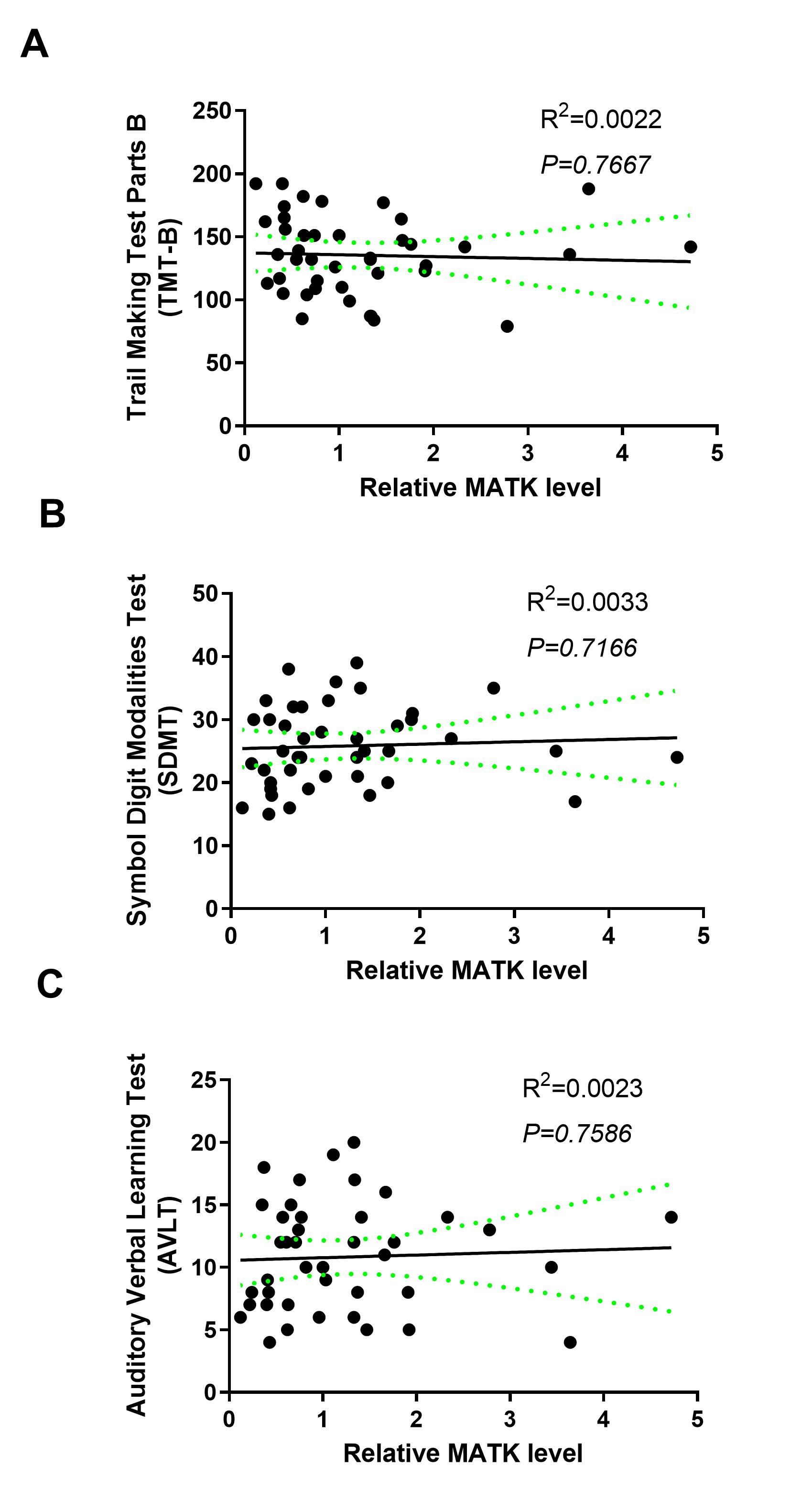

Supplement: Supplementary Figure 1 — Correlation of MATK with domain-specific neuropsychological tests. (A) Correlation of MATK with TMT-B. (B) Correlation of MATK with SDMT. (C) Correlation of MATK with SDMT. TMT, Trail Making Test Parts B; SDMT, Symbol Digit Modalities Test; AVLT, Auditory Verbal Learning Test. [file Image_1.jpeg]

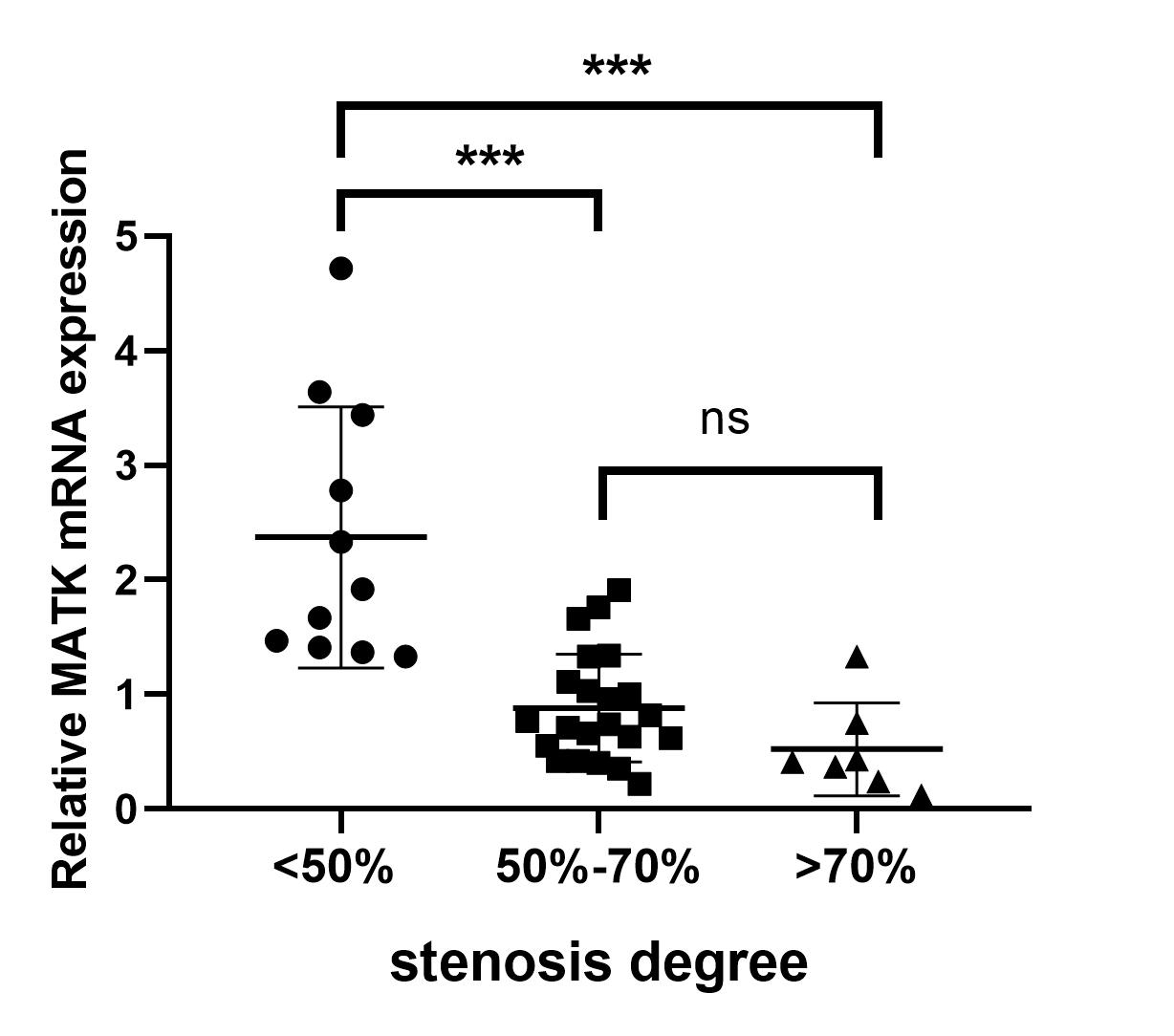

Supplement: Supplementary Figure 2 — Correlation of MATK with WMH patients’ cerebral artery stenosis. [file Image_2.jpeg]
